# Expressions of HIF-1α and MiR-210 in aqueous humor of patients with central retinal vein occlusion combined with macular edema

**DOI:** 10.12669/pjms.38.5.5092

**Published:** 2022

**Authors:** Pinghui Hu, Guanghui Liu, Huaping Sun, Wei Wei

**Affiliations:** 1 Pinghui Hu, Department of Ophthalmology, Nanjing University of Traditional Chinese Medicine, Li Xiang Eye Hospital of Soochow University, Suzhou 215000, Jiangsu, China; 2 Guanghui Liu, Department of Ophthalmology, People’s Hospital Affiliated to Fujian University of Traditional Chinese Medicine, Fuzhou 350004, Fujian, China; 3 Huaping Sun, Department of Ophthalmology, Nanjing University of Traditional Chinese Medicine, Jiangsu Provincial Hospital of Traditional Chinese Medicine, Nanjing 210029, Jiangsu, China; 4 Wei Wei, Department of Ophthalmology, Nanjing University of Traditional Chinese Medicine, Jiangsu Provincial Hospital of Traditional Chinese Medicine, Nanjing 210029, Jiangsu, China

**Keywords:** Central retinal vein occlusion, Macular edema, Aqueous humor, Hypoxia-inducible factor 1α, microRNA-210

## Abstract

**Objectives:**

To detect the expressions of hypoxia-inducible factor 1α (HIF-1α) and microRNA-210 (miR-210) in the aqueous humor of patients with central retinal vein occlusion (CRVO) combined with macular edema, and explore their correlations with CRVO combined with macular edema.

**Methods:**

A total of 65 patients (65 eyes) with CRVO combined with macular edema who were treated in the Lixiang Eye Hospital of Soochow University from April 2018 to March 2020 were selected as subjects (CRVO combined with macular edema group). Additionally, 74 patients (74 eyes) with cataract were selected during the same period as cataract control group. The expressions of HIF-1α mRNA and miR-210 in the aqueous humor were detected by quantitative real-time PCR (qRT-PCR). The levels of monocyte chemoattractant protein-1 (MCP-1), interleukin-6 (IL-6) and vascular endothelial growth factor (VEGF) in the aqueous humor were measured using enzyme-linked immunosorbent assay (ELISA). The correlations of HIF-1α mRNA and miR-210 in the aqueous humor of patients with CRVO combined with macular edema with vasoactive molecule levels, the diagnostic value of HIF-1α mRNA and miR-210 levels in the aqueous humor in CRVO combined with macular edema, as well as the factors influencing the occurrence of CRVO combined with macular edema were analyzed.

**Results:**

The levels of HIF-1α mRNA, miR-210, MCP-1, VEGF and IL-6 in the aqueous humor of the CRVO combined with macular edema group were higher than those of the cataract control group (*P* < 0.05). In the CRVO combined with macular edema group, HIF-1α mRNA and miR-210 levels in the aqueous humor were positively correlated (*r* = 0.522, *P* < 0.05), and they were positively correlated with MCP-1, VEGF and IL-6 levels (*P* < 0.05). The area under the curve (AUC) of HIF-1α mRNA and miR-210 in the aqueous humor in diagnosing CRVO combined with macular edema was 0.888 and 0.866, the specificity was 95.9% and 85.1%, and the sensitivity was 76.9% and 80.0%, respectively. The AUC of their combination was 0.937, with the specificity of 93.2% and the sensitivity of 86.2%. HIF-1α, miR-210 and VEGF were the independent risk factors affecting the occurrence of CRVO combined with macular edema (*P* < 0.05).

**Conclusion:**

In patients with CRVO combined with macular edema, HIF-1α mRNA and miR-210 were highly expressed in the aqueous humor, which may play an important role in the occurrence and development of the disease.

## INTRODUCTION

Central retinal vein occlusion (CRVO) is a common fundus vascular disease, and also one of the eye diseases that cause low vision and blindness. It often leads to macular edema, which is the main reason that CRVO affects the vision of patients.^1^,^2^ Consequently, it is very important to clarify the pathogenesis of CRVO combined with macular edema and realize early diagnosis. MicroRNA-210 (miR-210) is a member of miRNAs, which is closely related to the angiogenesis of endothelial cells after hypoxia.^3^,^4^ Hypoxia-inducible factor-1α (HIF-1α) is regulated by hypoxia signals, and its C-terminal is the active regulatory region, which can sense the hypoxia signals.^5^,^6^ Moreover, the retina of patients with CRVO may be hypoxic-ischemic. It is speculated that miR-210 and HIF-1α may be related to the pathogenesis of CRVO combined with macular edema.^7^ Therefore, in this study, the expression levels of miR-210 and HIF-1α in patients with CRVO combined with macular edema were detected, and their correlations with CRVO combined with macular edema were explored, so as to provide reference for controlling disease progression and improving the vision of patients.

## METHODS

A total of 65 patients (65 eyes) with CRVO combined with macular edema who were treated in the Lixiang Eye Hospital of Soochow University from April 2018 to March 2020 were selected as subjects (CRVO combined with macular edema group), including 30 males (30 eyes) and 35 females (35 eyes), with an average age of (50.33 ± 9.40) years. Additionally, 74 patients (74 eyes) with cataract were selected during the same period as cataract control group, including 34 males (34 eyes) and 40 females (40 eyes), with an average age of (50.47 ± 9.06) years. There were no statistically significant differences in age or gender between the two groups (*P* > 0.05).

### Ethical Approval:

The study was approved by the Institutional Ethics Committee of Nanjing University of Traditional Chinese Medicine, Li Xiang Eye Hospital of Soochow University on April 20, 2018 (No.201825), and written informed consent was obtained from all participants.

### Inclusion Criteria:

- CRVO patients with cystoid or diffuse macular edema confirmed by fundus fluorescein angiography and color fundus photography, with the results meeting the clinical diagnostic criteria for CRVO^8^;
- Patients approved by the Clinical Research Ethics Committee of the Lixiang Eye Hospital of Soochow University, and meeting ethical standards;
- Patients with complete medical records and willingness to participate in this study;
- Patients visiting for the first time, with the course of disease more than one month. ***Exclusion criteria:***
- Pregnant and lactating women;
- Patients who accompanied by systemic diseases affecting cytokines in the aqueous humor;
- Patients with history of other eye diseases and surgery.

### Main reagents and instruments:

MCP-1 ELISA kit (article No.: JK-a-H10038) was purchased from Shanghai Jingkang Bioengineering Co., Ltd. IL-6 ELISA kit (article No.: EGP0032) was purchased from Wuhan Fine Biotech Co., Ltd. VEGF ELISA kit (article No.: FB000103) was purchased from Beijing Futuae Biotech Co., Ltd. RNA extraction kit (article No.: R1200) and reverse transcription kit (article No.: RP1105) were purchased from Shanghai Hengfei Biotechnology Co., Ltd. miScript SYBR^®^ Green qPCR kit (article No.: 218076) was purchased from Applied Biosystems, USA. Microplate reader (type: AMR-100) was purchased from Hangzhou Allsheng Instruments Co., Ltd. qRT-PCR instrument (type: 7500) was purchased from Applied Biosystems, USA.

### Research Methods:

Sample collection and preservation: before intravitreal injection of ranibizumab, the aqueous humor of patients with CRVO combined with macular edema was slowly pumped out by puncture of the anterior chamber with a syringe needle. Before phacoemulsification and intraocular lens implantation, the aqueous humor was drawn out from the patients with cataract. One hundred μl aqueous humor was extracted from all patients. The collected aqueous humor was transferred to a microcentrifuge tube and stored in an ultra-low temperature freezer at - 80°C in the dark.

### Detection of vasoactive molecules:

The levels of monocyte chemoattractant protein-1 (MCP-1), interleukin-6 (IL-6) and vascular endothelial growth factor (VEGF) in the aqueous humor were measured using enzyme-linked immunosorbent assay (ELISA) in strict accordance with the manufacturer’s instructions.

### Detection of HIF-1α mRNA and miR-210 in the aqueous humor:

Total RNA was extracted from the aqueous humor using the RNA extraction kit, and the RNA was reversely transcribed into cDNA using the reverse transcription kit in strict accordance with the manufacturer’s instructions. HIF-1α, GAPDH, miR-210 and U6 were amplified using quantitative real-time PCR (qRT-PCR), and the primer sequences are shown in Table-I. The qRT-PCR system (a total of 20 μL) included 2 μL cDNA (50 ng/μL), 10 μL miScript SYBR® Green Mix, 0.8 μL upstream and downstream primers (10 μM) and 6.4 μL ddH2O. The primers were designed and synthesized by Shanghai Sangon Biotech Co., Ltd. The reaction conditions were as follows: pre-denaturation at 95°C for 5 min; 94°C for 30 s, 56°C for 30 s, 72°C for 45 s, totally 40 cycles. There were three replicates for each sample, and the relative expressions of HIF-1α mRNA and miR-210 in the aqueous humor were calculated using the 2^-∆∆CT^ method.

**Table I:** Primer sequences for qRT-PCR.

Gene	Forward primer 5’-3’	Reverse primer 5’-3’
HIF-1α	AAACAGAGCAGGTAATTGGAG	TCAAAGCGACAGATAACACG
GAPDH	AAGAGCTTGAAGTGGTGGT	CGCACACGGGAATAGGGCACG
miR-210	GTGCAGGGTCCGAGGT	TATCTGTGCGTGTGACAGCGGCT
U6	CTCGCTTCGGCAGCACA	AACGCTTCACGAATTTGCGT

### Statistical Analysis:

The data were statistically analyzed by SPSS 23.0. The measurement data were in a normal distribution and expressed as mean ± standard deviation (x̄±). The *t* test was used for their comparison between the two groups. The correlations of HIF-1α mRNA and miR-210 in the aqueous humor of patients with CRVO combined with macular edema with vasoactive molecule levels were analyzed by the Pearson method. The diagnostic value of HIF-1α mRNA and miR-210 in the aqueous humor in CRVO combined with macular edema was analyzed using the receiver operator characteristic (ROC) curve. The influencing factors for the occurrence of CRVO combined with macular edema were analyzed with a multivariate logistic regression analysis. *P* < 0.05 was considered as statistically significant.

## RESULTS

The levels of HIF-1α mRNA and miR-210 in the aqueous humor of the CRVO combined with macular edema group were higher than those of the cataract control group (*P* < 0.05). Table-II.

**Table II:** Comparison of HIF-1α mRNA and miR-210 levels in the aqueous humor between the two groups (χ¯±S).

Group	Cases (n)	HIF-1α mRNA	miR-210
Cataract control group	74	1.00 ± 0.30	1.00 ± 0.29
CRVO combined with macular edema group	65	1.72 ± 0.50	1.54 ± 0.35
*t*	-	10.435	9.944
*P*	-	0.000	0.000

Compared with the cataract control group, MCP-1, VEGF and IL-6 levels in the aqueous humor of the CRVO combined with macular edema group were higher (*P* < 0.05).Table-III.

**Table III:** Comparison of vasoactive molecule levels in the aqueous humor between the two groups (χ¯±S).

Group	Cases (n)	MCP-1 (pg/mL)	VEGF (pg/mL)	IL-6 (pg/mL)
Cataract control group	74	248.95 ± 52.73	146.37 ± 41.66	4.76 ± 1.23
CRVO combined with macular edema group	65	1126.84 ± 234.71	326.86 ± 49.52	162.58 ± 40.42
*t*	-	31.303	23.334	33.587
*P*	-	0.000	0.000	0.000

**Fig.1:**
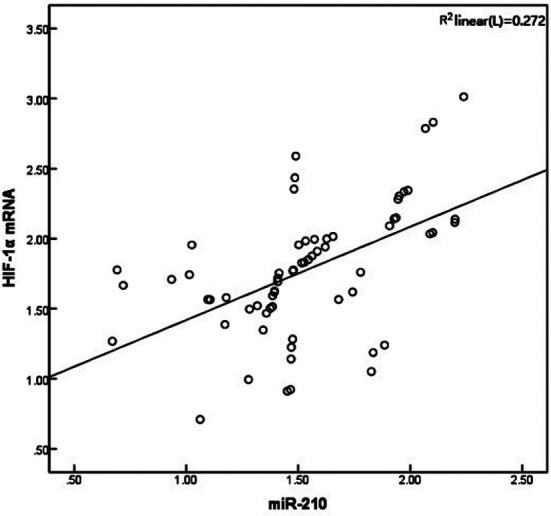
Correlation between HIF-1α mRNA and miR-210 in the aqueous humor of patients with CRVO combined with macular edema.

Pearson’s correlation analysis showed that in the CRVO combined with macular edema group, HIF-1α mRNA and miR-210 levels in the aqueous humor were positively correlated (*r* = 0.522, *P* < 0.05), and they were both positively correlated with MCP-1, VEGF and IL-6 levels (*P* < 0.05) (Table-IV, Fig.1).

**Table IV:** Correlations of HIF-1α mRNA and miR-210 in the aqueous humor of patients with CRVO combined with macular edema with vasoactive molecule levels.

Index		MCP-1	VEGF	IL-6
HIF-1α mRNA	*r*	0.492	0.521	0.493
	*P*	0.008	0.002	0.009
miR-210	*r*	0.504	0.517	0.486
	*P*	0.006	0.004	0.008

**Fig.2:**
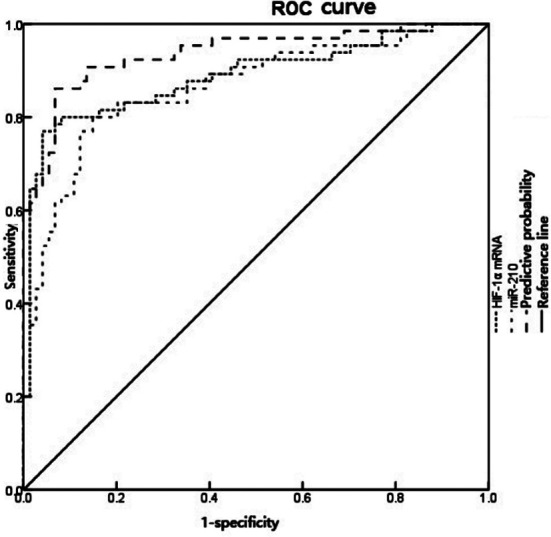
ROC curve for HIF-1α mRNA and miR-210 in the aqueous humor in diagnosing CRVO combined with macular edema.

The ROC curve was drawn with HIF-1α mRNA and miR-210 levels in the aqueous humor as test variables. The results demonstrated that the area under the curve (AUC) of HIF-1α mRNA and miR-210 in the aqueous humor in diagnosing CRVO combined with macular edema was 0.888 (95%CI: 0.829-0.947) and 0.866 (95%CI: 0.804-0.927), the cutoff value was 1.501 and 1.315, the specificity was 95.9% and 85.1%, and the sensitivity was 76.9% and 80.0%, respectively. The AUC of their combination was 0.937 (95%CI: 0.896-0.978), with the specificity of 93.2% and the sensitivity of 86.2% (Fig.2).Multivariate logistic regression analysis was carried out with CRVO combined with macular edema as the dependent variable, and HIF-1α, miR-210, MCP-1, VEGF and IL-6 levels as independent variables, revealing that HIF-1α, miR-210 and VEGF were the independent risk factors affecting the occurrence of CRVO combined with macular edema, as seen in Table-V (*P* < 0.05).

**Table V:** Multivariate logistic regression analysis of influencing factors for CRVO combined with macular edema.

Influencing factor	*B*	*SE*	*Wald*	*OR*	*95%CI*	*P*
HIF-1α	0.781	0.526	2.205	2.185	1.546~3.087	0.006
miR-210	0.665	0.458	2.108	1.945	1.316~2.876	0.007
MCP-1	0.186	0.245	0.576	1.205	0.513~2.829	0.164
VEGF	0.682	0.464	2.160	1.977	1.471~2.658	0.008
IL-6	0.353	0.348	1.029	1.424	0.672~3.016	0.096

## DISCUSSION

In clinic, CRVO is a common retinal vascular disease, which can lead to macular edema including cystoid edema.^9^,^10^ No capillary perfusion, tissue inflammation, ischemia and hypoxia can increase vascular permeability and further aggravate macular edema, and long-term diffuse macular edema can cause severe visual damage.^11^,^12^ Moreover, the treatment method of CRVO combined with macular edema is limited, and the efficacy is poor, which seriously affects the physical and mental health of patients.^13^ Therefore, it is urgent to explore relevant biological evaluation indexes for early detection and timely treatment in clinic, so as to improve the prognosis of patients with CRVO combined with macular edema.

HIF-1α is the most sensitive transcription factor to oxygen changes, and plays an important role in hypoxia-induced angiogenesis in retinal pigment epithelial cells.^14^,^15^ When the body is under hypoxia, HIF-1α binds to HIF-1β to form a compound, which can activate downstream target genes, and regulate the gene expression and protein synthesis of a series of vasoactive factors and cytokines.^16^ In this study, the results showed that HIF-1α mRNA level in the aqueous humor of patients with CRVO combined with macular edema was higher than that of patients with cataract, which is in line with the expression trend of HIF-1α in the study of Yan et al.^17^ Additionally, HIF-1α was an independent risk factor influencing the occurrence of CRVO combined with macular edema, suggesting that HIF-1α may play an important role in the pathogenesis of retinal hypoxia and ischemia in patients with CRVO combined with macular edema through high expression, resulting in the occurrence and development of the disease. Moreover, the AUC of HIF-1α mRNA in the aqueous humor in the diagnosis of CRVO combined with macular edema was 0.888, with the cutoff value of 1.501 and high diagnostic specificity (95.9%), which indicates that the risk of CRVO combined with macular edema is high when HIF-1α mRNA level > 1.501.

MiR-210 is a hypoxia-inducing miRNA, which is involved in the angiogenesis of visceral tissues under hypoxia-ischemia, and is closely related to blood supply and neuron protection.^18^ Under hypoxia, miR-210 is up-regulated in endothelial cells, which can regulate the expression of VEGF and promote the formation of capillary-like structures.^19^ Our study demonstrated that the miR-210 level in the aqueous humor of patients with CRVO combined with macular edema was higher than that of patients with cataract, which suggested that the high expression of miR-210 may be involved in retinal angiogenesis in patients with CRVO combined with macular edema, thus accelerating the development of the disease. VEGF can promote angiogenesis, increase vascular permeability and destroy the blood-retinal barrier.^20^ IL-6, an inflammatory response factors, can increase capillary permeability by regulating the expression of VEGF.^21^ MCP-1 is closely related to inflammatory diseases, which can induce the activation of monocytes and the chemotaxis of various immune response-related factors and inflammatory factors.^22^ In our study, it was found that the levels of MCP-1, VEGF and IL-6 in the aqueous humor of patients with CRVO combined with macular edema were higher than those of patients with cataract, and their expression were all positively correlated with HIF-1α mRNA and miR-210. Thereby, it was speculated that the expressions of HIF-1α mRNA and miR-210 were increased in patients with CRVO combined with macular edema due to retinal ischemia and hypoxia, and miR-210 could affect the sensitivity of VEGF^23^, so that its expression was increased, thus damaging the blood-retinal barrier, increasing vascular permeability and further causing the aggravation of macular edema. Furthermore, the AUC of miR-210 in the aqueous humor in diagnosing CRVO combined with macular edema was 0.866, which was smaller than HIF-1α mRNA. Nevertheless, its diagnostic sensitivity was high (80.0%). What is more, the AUC and sensitivity of their combination were enhance, indicating that in clinical practice, they can be considered as reference indexes for combined use to evaluate CRVO combined with macular edema.

### Limitations of the study:

In this study, only HIF-1α and miR-210 contents were detected in the aqueous humor, which cannot represent the contents in the retinal tissue and vitreous cavity. Therefore, there are some limitations, which need to be confirmed by further basic experiments in the future. In addition, this study was a descriptive study, which only discussed biomarkers conducive to the early clinical detection of CRVO combined with macular edema, and did not discuss the direct treatment plan for CRVO combined with macular edema patients. We look forward to exploring therapeutic options that may help patients with CRVO combined with macular edema in future studies.

## CONCLUSION

In patients with CRVO combined with macular edema, HIF-1α and miR-210 were highly effective in the aqueous humor and positively correlated. They may be synergistically involved in the occurrence and development of the disease. In clinical practice, they can be used as reference indexes to evaluate and diagnose CRVO combined with macular edema.

### Authors’ Contributions:

**PH & GL:** Designed this study ,prepared this manuscript, are responsible and accountable for the accuracy and integrity of the work.

**WW:** Collected and analyzed clinical data.

**HS:** Significantly revised this manuscript.s
